# Schistosomiasis in the Democratic Republic of Congo: a literature review

**DOI:** 10.1186/s13071-015-1206-6

**Published:** 2015-11-19

**Authors:** Joule Madinga, Sylvie Linsuke, Liliane Mpabanzi, Lynn Meurs, Kirezi Kanobana, Niko Speybroeck, Pascal Lutumba, Katja Polman

**Affiliations:** Institute of Health and Society, Université Catholique de Louvain, Brussels, Belgium; Department of Biomedical Sciences, Institute of Tropical Medicine, Antwerp, Belgium; Institut National de Recherche Biomédicale, Kinshasa, Democratic Republic of Congo; Department of Tropical Medicine, University of Kinshasa, Kinshasa, Democratic Republic of Congo

## Abstract

Schistosomiasis is a poverty-related parasitic infection, leading to chronic ill-health. For more than a century, schistosomiasis has been known to be endemic in certain provinces of the Democratic Republic of Congo (DRC). However, a clear overview on the status of the disease within the country is currently lacking, which is seriously hampering control. Here, we review the available information on schistosomiasis in DRC of the past 60 years. Findings and data gaps are discussed in the perspective of upcoming control activities.

An electronic literature search via PubMed complemented by manual search of non-peer-reviewed articles was conducted up to January 2015. The search concerned all relevant records related to schistosomiasis in the DRC from January 1955 onwards. A total of 155 records were found, of which 30 met the inclusion criteria. Results were summarized by geographical region, mapped, and compared with those reported sixty years ago. The available data reported schistosomiasis in some areas located in 10 of the 11 provinces of DRC. Three species of *Schistosoma* were found: *S. mansoni*, *S. haematobium* and *S. intercalatum*. The prevalence of schistosomiasis varied greatly between regions and between villages, with high values of up to 95 % observed in some communities. The overall trend over 60 years points to the spread of schistosomiasis to formerly non-endemic areas. The prevalence of schistosomiasis has increased in rural endemic areas and decreased in urban/peri-urban endemic areas of Kinshasa. Hepatosplenomegaly, urinary tract lesions and anaemia were commonly reported in schistosomiasis endemic areas but not always associated with infection status.

The present review confirms that schistosomiasis is still endemic in DRC. However, available data are scattered across time and space and studies lack methodological uniformity, hampering a reliable estimation of the current status of schistosomiasis in DRC. There is a clear need for updated prevalence data and well-designed studies on the epidemiology and transmission of schistosomiasis in DRC. This will aid the national control program to adequately design and implement strategies for sustainable and comprehensive control of schistosomiasis throughout the country.

## Background

Schistosomiasis is a common parasitic disease caused by worms belonging to the genus *Schistosoma*. This neglected tropical disease (NTD) prevails mainly in poor areas of tropical and sub-tropical countries, where access to safe drinking water and adequate sanitation is lacking [1]. Parasite transmission requires contamination of surface water by eggs contained in human excreta, presence of specific freshwater snails (intermediate host) and human contact with infested water [2]. Around 230 million people are infected worldwide, of whom more than 90 % live in sub-Saharan Africa [3, 4]. The two major schistosome species infecting man are *S. mansoni*, which is transmitted by *Biomphalaria* snails, and *S. haematobium*, transmitted by *Bulinus* snails. The geographical distribution of the different species depends on the ecology of the snail hosts. Natural streams, ponds, and lakes as well as artificial waters such as dams and irrigation canals are typical sources of infection [5]. Chronic disease is mainly caused by immunopathological reactions against schistosome eggs trapped in the intestines or liver (for *S. mansoni)* or in the bladder and urogenital system (for *S. haematobium*), leading to organ-specific effects such as hepatosplenic inflammation and liver fibrosis, and inflammatory and obstructive disease of the urinary tract [6]. Schistosomiasis is also a recognized cause of anaemia [7, 8], stunting [9] and impaired cognition [10]. Other species infecting man in Africa are *Schistosoma intercalatum* and *Schistosoma guineensis*, but these are of relatively minor public health importance [11]. Currently, the main control strategy against schistosomiasis is regular population-based anthelminthic treatment with praziquantel (PZQ), aiming at reducing current infection and preventing the development of severe disease in specific risk groups (predominantly school-age children) [12].

The Democratic Republic of Congo (DRC) is the second largest African country (2,345,400 km^2^), situated in the heart of Africa and sharing boundaries with nine neighbouring countries. The country, which has faced decades of war, is one of the poorest countries worldwide, with 50 % of the population living without access to safe water and sanitation [13]. Schistosomiasis has been known to be present in certain provinces of DRC for more than a century [14]. After the first cases of schistosomiasis were detected in DRC in 1897, surveys have been conducted in several areas, and data were collected during colonial times (1908–1960). These early reports were reviewed in 1954 by Gillet and Wolfs [15]. The country achieved independence on June 30th 1960. Since then, there has been no clear overview on the status of schistosomiasis within the country. In 2012, the Ministry of Health adopted a national plan against neglected tropical diseases (NTDs), including schistosomiasis [16]. Future school-based mass treatment campaigns for five NTDs including schistosomiasis are now gradually being implemented throughout the country. The purpose of this review is to summarize the information currently available on schistosomiasis in the country and to identify knowledge gaps that need to be addressed in the perspective of upcoming control activities.

## Methods

### Literature search strategy

**Fig. 1:**
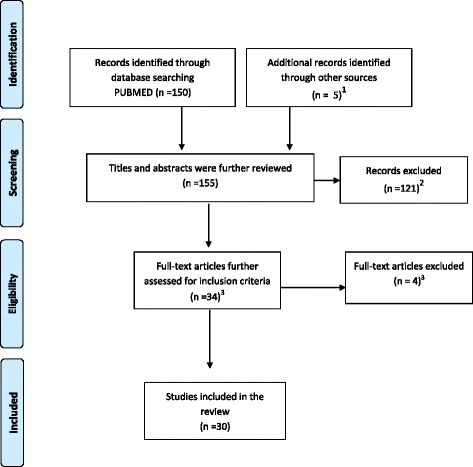
Flow diagram of the literature search strategy. 1. Additional records consisted of three master theses, one book chapter and one map of schistosomiasis in DRC. 2. Reasons for exclusion were: a) Non relevant association between the keywords (e.g., Congo as name of a reagent, another country “Congo-Brazzaville”, etc.) (20 records); b) Reviews and studies based on mathematical modelling (18 records); c) Case reports and studies on hospitalized patients without any information on their geographic residency (11 records); d) Cases of schistosomiasis among non-Congolese travelers coming from DRC (5 records); e) Malacological studies (11); f) Animal and parasite fundamental research (30 records); g) Test evaluation and others (32 records). 3. Reason for exclusion: duplicates (4 records)

A literature search was conducted in PubMed to identify relevant original articles related to schistosomiasis in the DRC, published between January 1955 (i.e. after the last review of Gillet et al. in 1954 [15]) and January 2015. The search strategy used the keywords “Schistosoma” or “schistosomiasis” and “Congo” or “Zaïre”. The search was limited neither by language nor by study design. Bibliographies of published studies were screened to find additional sources of data. In addition, a manual search was performed to find unpublished data and theses. Titles and abstracts of records were screened using the following inclusion criteria: (i) Reporting presence of schistosomiasis in an area of DRC and/or (ii) Reporting prevalence/frequency of schistosomiasis in an area of DRC and/or (iii) Reporting schistosomiasis-related morbidity in an area of DRC and/or (iv) Reporting schistosomiasis control in an area of DRC. For all abstracts meeting inclusion criteria, full papers were retrieved and further screened. Duplicates were excluded. The flow diagram of the literature search strategy is shown in Fig. 1.

### Retrieved data

From the studies fulfilling the inclusion criteria, the following data were recorded: first author, year of publication, study design, name of the locality or area, study population, number of people tested, diagnostic method, species of *Schistosoma* detected, prevalence of infection, population at risk, infection intensity, schistosomiasis-related morbidity and schistosomiasis control activities.

### Mapping

**Fig. 2:**
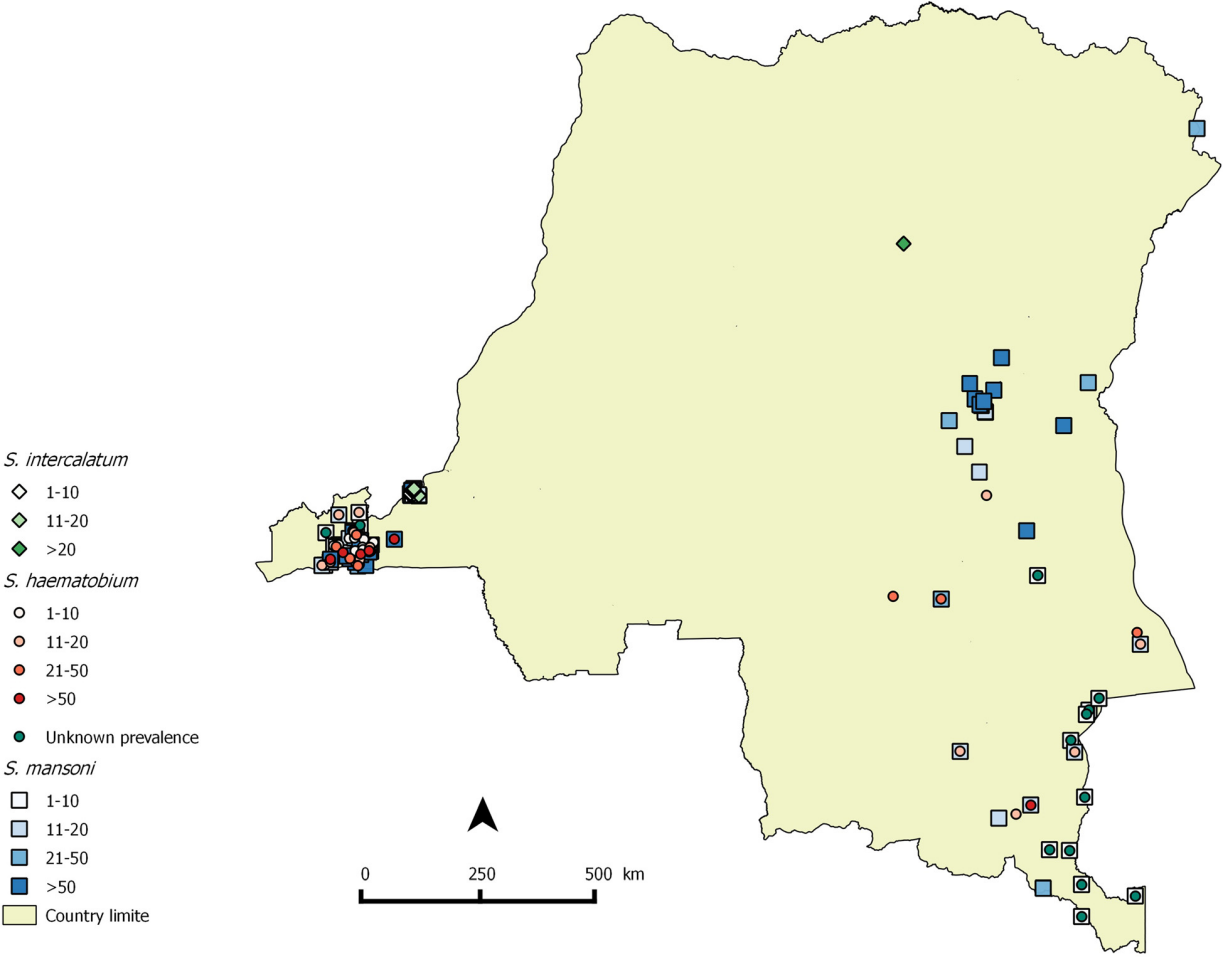
Distribution map of schistosomiasis in DRC in 1954. Of the total number of 66 survey locations reported, 46 could be mapped; for the other locations geographical coordinates were lacking

**Fig. 3:**
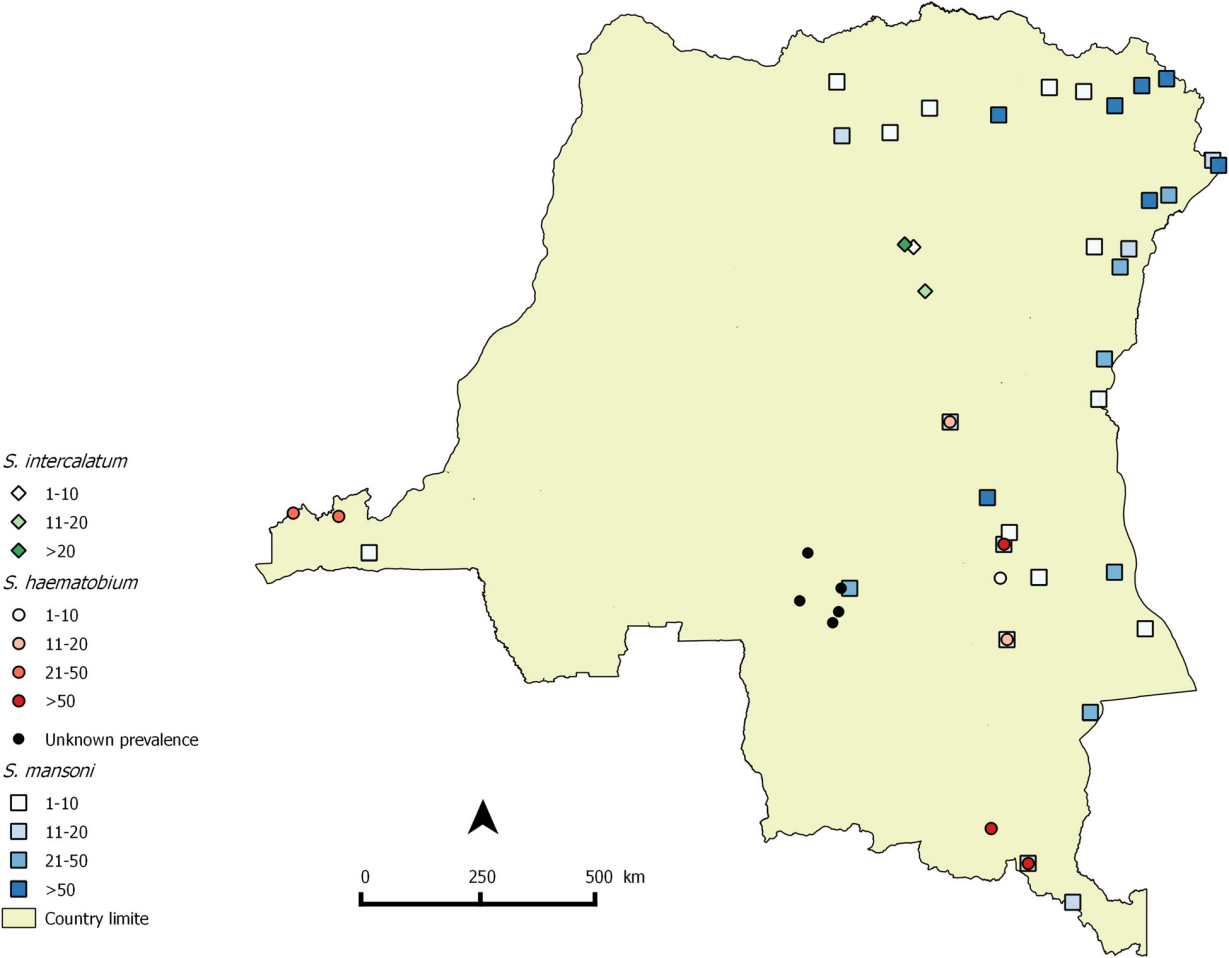
Distribution map of schistosomiasis in DRC based on reports from 1955–2015. Of the total number of 389 survey locations reported, 234 could be mapped; for the other locations geographical coordinates were lacking

Geographic coordinates of survey locations reported by Gillet & Wolfs [15] and those retrieved from the studies in the present review were searched in the database of the “Observatoire Satelitale des Forêts d’Afrique Centrale” (OSFAC), Kinshasa, DRC. Subsequently, these were linked to the respective prevalences reported for that location. Then, distribution maps of schistosomiasis in DRC in 1954 and in the past 60 years were drawn using QGIS 2.2.0 (Figs. 2 and 3).

## Results

### Characteristics of retrieved records

From a total of 155 records found, 30 records met the inclusion criteria and were included in the present review; of these, 28 were peer reviewed publications and two were unpublished data. Six records were published between 1955 and 1974, 17 records between 1975 and 1994, and seven in the last twenty years. The prevalences of *Schistosoma* infection are summarized in Table 1 and prevalence of schistosomiasis-related morbidity in Table 2.

**Table 1:** Overview of reports on human infections with *Schistosoma* spp. in DRC

Province	Location	Geographic unit	Year of publication	Study population	Number of people tested (n)	Species	Infection prevalence (%)	Risk group	Diagnostic method used	References
Kinshasa	Kintambo	1 school	1976	SAC	50	Sm	4.0	NR	Coprology + rectal snip	[17]
	Kitambo and Bandalungua	NR	1977	TP	1,818	Sm	16.3	12–15 years	Ritchie	[18]
	Kitambo and Bandalungua	NR	1983	SAC	735	Sm	39.6	Males 13–14 years	Kato	[21]
	Quartier Brikin	NR	1987	TP	156	Si	30.0	10–19 years	Kato	[20]
	Mangungu and Tsudi rivers area	NR	1997	SAC	167	Si	3.6	NR	Kato-Katz	[25]
	Random selection	26 schools	2009	SAC	1,559	Sm	3.1	NR	Kato-Katz	[Linsuke: Schistosomiasis in schoolchildren of Kinshasa and Bas-Congo provinces, Democratic Republic of Congo, unpublished]
						Sh	0.13	NR	Stick test	
						Si	0.6	NR	Kato-Katz	
	Mokali health area	2 schools	2014	SAC	616	Sm	6.4	Girls	Kato-Katz	[24]
Bas-Congo	Konde-kuimba	1 village	1985	TP	510	Sm	63.0	Males 10–19 years	Kato-Katz	[19]
								Females 5–19 years		
								Palm oil extractors		
	Songololo territory	57 schools	2000	SAC	5,806	Sm	31.2	NR	Kato-Katz	[22]
					2,495	Sh	20.2	NR	Stick test	
	Kimpese and Nsona- Mpangu health districts	26 schools	2009	SAC	840	Sm	25.2	NR	Kato Katz	[Linsuke: Schistosomiasis in schoolchildren of Kinshasa and Bas-Congo provinces, Democratic Republic of Congo, unpublished]
						Sh	10.0	NR	Urine sedimentation	
Eastern Kasaï	Kasansa health district	6 schools	2014	SAC	335	Sm	82.7	Male	Kato-Katz	[26]
Maniema	SOMINKI mining zone	2 villages	1987	TP	910	Sm	19.0 and 96.0	Subjects under 18 years	Kato	[30]
	SOMINKI mining zone	10 villages	1986	TP	6,433	Sm	73.0–96.0	NR	Kato	[36]
	SOMINKI mining zone	38 villages	1985	TP	8,958	Sm	7.1–96.7	11–20 years	Kato	[34]
	SOMINKI mining zone	5 villages	1989	TP	4,073	Sm	>80.0	11–20 years	Kato	[31]
	SOMINKI mining zone	4 village	1984	male labourers 22–35 years	154	Sm	3.0–93.0	NR	Kato	[38]
	Kasongo	1 school	1981	SAC	61	Sh	6.6	NR	Sedimentation	[29]
					52	Sm	71.1	NR	Kato-Katz	
	Kindu	1 school	1981	SAC	32	Sh	72.0	NR	Sedimentation	[29]
					40	Sm	17.5	NR	Kato-Katz	
South-Kivu	Katana	>3 villages	2000	TP	787	Sm	8.1	10–14 years	Kato-Katz	[28]
Eastern province	Yakusu	NR	1956	TP	470	Si	38.6	Children and adolescents	Coprology	[46]
	Aru territory	3 schools	1986	SAC	1,550	Sm	12.4–21.2	6–15years	Ritchie and Kato-Katz	[47]
Katanga	Lac Lufira reservoir area	77 villages	1969	TP	3,019	Sh	12.1	10–14 years	Urine sedimentation	[43]
					3,019	Sm	6.3		Willis + Telleman Bailenger	

*Abbreviations*: *SOMINKI* Société Minière du Kivu; *Sm Schistosoma mansoni*; *Sh S. haematobium*; *Si S. intercalatum*; *TP* total population; *SAC* school-aged children; *NR* not reported

**Table 2:** Overview of reports on schistosomiasis-related morbidity in DRC

Province	Locality/area	Study design	Study population	Hepatomegaly prevalence (%)	Splenomegaly prevalence (%)	Urinary tract lesion prevalence (%)	Anaemia prevalence (%)	References
Kinshasa	Mokali health area	Cross-sectional	SAC	–	–	–	41.6	[24]
Bas-Congo	Konde-Kuimba	Cross-sectional	TP	40.0–54.0	8.0–22.0	–	–	[19]
Maniema	Kindu and Kasongo	Cross-sectional	SAC	11.0	0.5	–	–	[29]
	SOMINKI mining area	Cross-sectional	IP	3.0–45.0	9.0–22.0	–	9.0–36.0	[30]
	SOMINKI mining area	Cohort	IP	15.8–28.8	6.4–29.0	–	–	[37]
Katanga	Lac Lufira	Cross-sectional	IP	–	–	8–60	–	[41]
Eastern province	Aru territory	Cross-sectional	SAC	15.6–38.0	22.0–59.2	–	–	[47]
Katanga	Lac Lufira area	Cross-sectional	IP	29.5	34.4	–	21.6	[42]
Eastern Kasaï	Kasansa health district	Cross-sectional	SAC	–	–	–	61.4	[27]

*Abbreviations*: *SOMINKI* Société Minière du Kivu; *TP* total population; *SAC* school-age children; *IP* infected people

### Distribution of *Schistosoma* spp. infection and related morbidity

Three species of *Schistosoma* were found: *S. mansoni*, *S. haematobium* and *S. intercalatum*. Combining our review data with those reviewed by Gillet & Wolfs [15], it appears that schistosomiasis endemic areas are present in 10 of the 11 provinces of DRC. Below, we discuss the main results by geographical region following the example of Gillet & Wolfs [15].

#### Western region

This region extends from Kinshasa to the Atlantic Ocean and contains the transmission areas of Kinshasa and Bas-Congo provinces. We identified ten records reporting on schistosomiasis in this region in the past sixty years [17–25].

In Kinshasa, the capital city of the country, schistosomiasis was not endemic before 1954 [15]. The first study reporting possible local transmission of schistosomiasis in Kinshasa was published in 1976, based on a combination of malacological, clinical, biological and epidemiological investigations [17]. An *S. mansoni* infection prevalence of 4 % was reported in 50 schoolchildren living along the River Basoko and its tributaries, where suspected snail vectors were found. The presence of this small native focus was confirmed a year later, by a population-wide study carried out in the same area, with a prevalence of 16.3 % [18]. Five years later, another study in this urban schistosomiasis focus reported a 39.6 % *S. mansoni* infection prevalence among schoolchildren [21]. The infection spread was limited to the districts of Bandalungwa and Kintambo, and the transmission was reported to be irregular, because of the seasonal disappearance of snail vectors. The first study reporting *S. intercalatum* infection in Kinshasa was published in 1987 [20]. The infection was reported to be present in Brikin, a city in the west periphery of Kinshasa where a prevalence of 30 % was observed. The authors also mentioned two cases of *S. haematobium* infection imported from the neighbouring province of Bas-Congo. A decade later, a combined epidemiological and malacological study in the same area revealed a decrease of the human *S. intercalatum* infection prevalence to 3.6 %, probably due to changes in socio-economic status and irregular transmission [25]. In 2009, a parasitological survey in randomly selected schools of Kinshasa found an overall prevalence of schistosomiasis infection of 3.4 % among schoolchildren (*n* = 1,559), with prevalences of 3.1, 0.13 and 0.6 % for *S. mansoni*, *S. haematobium* and *S. intercalatum*, respectively [Linsuke: Schistosomiasis in schoolchildren of Kinshasa and Bas-Congo provinces, Democratic Republic of Congo, unpublished]. Recently in 2014, a prevalence of 6.4 % for *S. mansoni* was found in 616 schoolchildren from two primary schools of Mokali, a rural health area of Kinshasa [24]. Anaemia was reported in 41.6 % of the study population, and showed a significant association with *S. mansoni* infection.

In contrast to Kinshasa, Bas-Congo province was already endemic for schistosomiasis before 1954. Two foci of *S. mansoni* (Kimpese and Buku-Bandu) and two foci of *S. haematobium* (Buku-Dunji and the island of Mateba) have been described [15]. In 1951, as a result of treatment, schistosomiasis prevalence was low, ranging from 0.6 to 4.1 % [15]. Twenty years later, however, in 1974, Mandahl Barth et al. [23], reported 22 *S. mansoni* and *S. haematobium* endemic communities in this province, though the authors did not provide prevalence data. Further to the west, De Clercq et al. [19] reported a *S. mansoni* infection prevalence of 63 % in 1985, with moderate infection intensities [mean egg count of 189 eggs/gram of stool (epg)] in the population of Konde-Kuimba, a village in Mayombe land. Prevalence of hepatomegaly ranged from 40 to 54 %, while for splenomegaly, prevalence ranged from 8 to 22 %. Associations between hepatosplenomegaly and infection status were not assessed. More recently, a survey conducted in 2000 reported a *S. mansoni* prevalence of 31.2 % (0–89 %) in schoolchildren of Songololo territory, which includes the previously reported schistosomiasis endemic focus of Kimpese and its neighbouring health district of Nsona-Mpangu [22]. A parasitological survey conducted in randomly selected schools from the same area in 2009 (n = 840) reported a 32.1 % schistosomiasis prevalence, again with a large range (0–84 %) between schools [Linsuke: Schistosomiasis in schoolchildren of Kinshasa and Bas-Congo provinces, Democratic Republic of Congo, unpublished].

#### Central region

The central region covers the Eastern and Western Kasaï provinces. Data collected in this region before 1954 reported *S. mansoni* infection in communities between Rivers Lubilash and Mbuji-Mayi (Eastern Kasaï) as well as in areas surrounding Lake Fwa (Western Kasaï), with a prevalence up to 80 and 100 % in some communities. Treatment campaigns were conducted from 1946 to 1950 and schistosomiasis prevalence reduced, ranging from 6.8 to 64 % between villages [15].

During the past sixty years, only one more study has been conducted on schistosomiasis in this region [26, 27]. The study was conducted in 2011, among schoolchildren of the Kasansa health district (Eastern Kasaï province) and reported an overall *S. mansoni* prevalence of 82.7 %, ranging from 59.9 to 94.9 % between schools. Moreover, 41.1 % of the sampled population presented high intensity infections (mean egg count > 400 epg) [26]. Anaemia was prevalent in 61.4 % of schoolchildren but no significant association was found with *S. mansoni* infection [27]. No information on schistosomiasis in Western Kasaï province has been published during the last sixty years.

#### Eastern region

This region consists of the Maniema, North Kivu and South Kivu provinces. During the last sixty years, 11 records were published based on data from this region [28–38].

In the Maniema province, two sub-areas can be distinguished. The first sub-area lies along the Congo River. Two cities of this sub-area (Kindu and Kasongo) have been studied over time. In 1950, a high prevalence of *S. mansoni* infection (80 %) was reported among schoolchildren of Kasongo, while no *S. haematobium* infection was found. At the same time, in Kindu, prevalences of 5.5 and 9 % were reported, for *S. haematobium* and *S. intercalatum* respectively, with five imported cases of *S. mansoni* infection [15]. Three decades later, in 1981, a parasitological survey was conducted among schoolchildren from the same cities [29]. In Kasongo, *S. mansoni* prevalence remained high (71.1 %) and *S. haematobium* appeared to emerge (6.6 %). In Kindu, *S. haematobium* prevalence increased (up to 72 %), and *S. mansoni* infection appeared to emerge (17.5 %), while *S. intercalatum* cases were not found anymore. Among the pupils examined, 11 and 25 % had moderate liver enlargement while 0.5 and 1.1 % had enlarged spleens respectively. No association was found between these indices and *S. mansoni* infection intensity.

The second sub-area is a tin mining zone characterized by an extensive system of artificial lakes and water drainage, described by Polderman et al. [35, 36]. Only *S. mansoni* has been reported in this sub-area, with high infection prevalences (up to 96.7 %), and 11–20 year old individuals carrying the highest infection intensities (range: 648–1,551 epg) [31, 33]. This high level of endemicity was attributed to extensive water works as well as armed conflicts of 1964, which stopped detection and treatment of infected mine workers and allowed migration to this area of people from heavily infected regions [35, 36]. In the same area, a study compared the prevalence of schistosomiasis-related morbidity between two villages, Makundju and Masimelo [30]. The first was considered a high and the second a low *S. mansoni*-endemic village, based on schistosome infection prevalences published in a previous study (*S. mansoni* infection prevalence of 80 and 12 %, respectively). Schistosomiasis-related morbidity was more pronounced in Makundju, with 45 % of the population presenting with enlargement of the left liver lobe, 32 % with right lobe hepatomegaly, and 29 % with splenomegaly. In Masimelo, these percentages were 9, 3 and 9 %, respectively. Anaemia was found in 30 % of males and 21 % of females in Makundju and in 9 % of males and 36 % of females in Masimelo. From 1978 onwards, heavily infected people (>600 epg) were treated annually in this area, using hycanthone, then oxaminique and finally praziquantel combined with focal molluscicidal treatment [39, 40]. After 8 years of intervention, prevalence and intensity of infection were almost the same as before control while hepatomegaly and to a lesser extent, splenomegaly had dramatically decreased [37].

In North Kivu province and until 1954, *S. mansoni* infection was reported in areas along the left bank of Lake Edward and its tributaries, from Beni and Butembo to the border with South Kivu province. Prevalence was higher among mining workers (27 %) than in the total population (<13 %) [15]. No data have been published from this province during the past sixty years.

In South Kivu province, areas located along the left bank of Lake Kivu have been reported to be endemic for *S. mansoni* during colonial times. Bobandana, Bukavu and Ngombo island were the main foci with infection prevalences of 22.7, 8.84 and 19.1 %, respectively [15]. In 2000, an *S. mansoni* infection prevalence of 8.1 % was reported in new villages built around a network of fish ponds close to Katana, a city lying close to Bukavu [28].

#### South-eastern region

The south-eastern region encompasses the Katanga province where both *S. mansoni* and *S. haematobium* were reported during colonial times. Schistosomiasis was reported in towns such as Lubumbashi, Likasi and Sakania, including their surrounding agglomerations. Both species were reported to be present along the Lualaba River, from the center to the northern boundary of the province. Infections with *S. mansoni* alone were mentioned in the East of the province, in communities living on the left bank of Lake Tanganyika (30 % in Kalemie and 8 % in Moba) and Lake Moero (27 % in Luanza) and, at the West of the province, in the territory of Kaniama, close to the boundary with Eastern Kasaï province [15].

In the early 1970s, a new focus for both *S. mansoni* and *S. haematobium* comprising 54 villages was described around the artificial lake Lufira [41–45]. An overall prevalence of 6.3 and 12.1 % for *S. mansoni* and *S. haematobium*, respectively, were reported [43]. Overall prevalence of clinical anaemia was 21.6 %, while hepatomegaly and splenomegaly were present in 29.5 and 34.4 % of infected people [3]. In two villages (i.e. Kapolowe and Lupidi), endoscopic and radiographic examinations of *S. haematobium*-infected people showed prevalences of urinary tract lesions ranging from 8–60 % depending on the age category. The lesions included severe cystoscopic lesions (60 %), bladder calcification (26 %), pyelonephritis-like lesions (8 %), unilateral ureteral dilatations (13 %) and bilateral ureteral dilatations (7 %) [41].

#### Northern region

The Northern region contains the Eastern and Equator provinces. Three endemic sub-areas were described in Eastern province before 1954. The first lies on the left bank of Lake Albert where *S. mansoni* infection was reported with prevalences ranging from 11 % in Mokambo to 64.9 % in Kasenyi. The second lies along the Uélé River and its tributaries (Kibali and Bomokandi) with *S. mansoni* prevalences ranging from 1.2 % in Dungo to 92.6 % in Aba. The third is situated in the district of Kisangani, where only *S. intercalatum* was endemic with a 2.8 % prevalence in Kisangani and 72–79 % in a 5–30 year-old male population in Yakusu [15]. In the province of Equator, *S. mansoni* infection was reported along the Ubangui River with prevalences of 0.3 and 0.6 % in Mobayi and Mogoro respectively, while *S. intercalatum* infection was reported in Lukandu (0.6 %).

Among the aforementioned areas, only Yakusu has been surveyed during the past sixty years (in 1956), and a *S. intercalatum* infection prevalence of 38.6 % was reported [46]. Another study was conducted in 1986 among children and adolescents (6–20 years) of three villages of Aru region where up to 70,000 refugees immigrated from Uganda in 1979 [47]. An *S. mansoni* prevalence of 10.8 to 31.5 % was reported, with 7.9 % of the study population excreting more than 100 epg. The study reported prevalences of 15.6 to 38 % for hepatomegaly and of 22 to 59.2 % for splenomegaly. No significant association was found between hepatosplenomegaly and schistosome infection status. No data on schistosomiasis have been generated in the Equator Province in the last 60 years.

## Discussion

The objective of the present review was to summarize available epidemiological data on schistosomiasis in DRC and to discuss findings and knowledge gaps in view of control. Data reviewed here indicate that schistosomiasis is still endemic in DRC. However, it has received little attention in the last sixty years and many questions on its epidemiology in the country remain unanswered.

The overall trend points to the spread of schistosomiasis to formerly non-endemic areas, an increase in prevalence in rural endemic areas and a decrease in urban/peri-urban endemic areas of Kinshasa. However, in-depth analysis of area-specific prevalences over time reveals more complex trends. The persistence of schistosomiasis in formerly endemic areas and the onset of new transmission areas may be (partly) explained by the absence of control activities, low access to safe water, and human migration. Indeed, the only control activities that have been performed in DRC during the last 60 years took place in Maniema province and targeted only heavily infected individuals [37]. Water access rates in DRC have undergone a dramatic decline during the 90s [48] and lie currently around 50 %, with a great disparity between urban and rural areas [13]. Significant population movements in recent conflicts may have introduced the disease in new areas. Indeed, 2.4 million people were displaced within DRC because of the war and 46,300 refugees came from neighbouring and endemic countries [49]. These and other parameters are likely to have contributed to the fact that schistosomiasis has (re-)emerged in the country, or is at risk of doing so along new patterns. Much remains to be studied about transmission patterns of schistosomiasis in DRC; so far only four studies [17, 18, 20, 25] have combined human schistosomiasis surveys with malacological studies.

Hepatosplenomegaly, anaemia, and urinary tract lesions were reported in some schistosomiasis endemic areas, but they were not always statistically associated with schistosome infection. The absence of significant associations between *S. mansoni* infection and hepatosplenomegaly or anaemia could be partly due to co-endemicity with malaria, soil-transmitted helminth infections, malnutrition and other diseases leading to the similar clinical disorders and acting as confounders [50]. Also, detection methods and definitions of clinical hepatomegaly and other disease outcomes varied considerably between studies, not allowing reliable spatio-temporal comparisons or good evaluation of the disease burden in affected areas. WHO standardized tools for measurement of schistomiasis-related organ lesions [51], anaemia [52, 53] and stunting [54] should be applied in future studies, to overcome the subjectivity inherent to clinical measurements.

## Conclusion

Since the first cases of schistosomiasis were reported in the DRC in 1897, data have been accumulated by the health services during colonial times, showing a widespread distribution of this disease in the country, as reviewed in 1954 by Gillet & Wolfs [15]. During the past 60 years, however, information has been scarce and scattered across time and space, hampering a reliable estimation of the current status of schistosomiasis in DRC. Overall, the currently available studies, as presented in this review, are limited, often outdated and lacking methodological uniformity. For the major part of the DRC, there are no data on schistosomiasis and it is thus unknown whether the disease is absent in these regions or, more likely, simply not measured. Nevertheless, considering the extremely high prevalence of infection observed in some foci by recent studies, there is no doubt that schistosomiasis represents a major public health concern in DRC. There is an urgent need for updated prevalence data and well-designed studies on the epidemiology and transmission of this important NTD. This review will be of use to the national program for NTD control and provide a good basis to inform, and prioritize, schistosomiasis prevalence mapping and upcoming control activities.
